# Long-Term Disease Control of Locally Invasive Epithelial-Myoepithelial Carcinoma of Parapharyngeal Salivary Glands With Definitive Radiotherapy

**DOI:** 10.7759/cureus.42669

**Published:** 2023-07-29

**Authors:** Adeel Riaz, Taskheer Abbas, Syed Mohsin Raza, Abu Hurrairah, Arif Jamshed

**Affiliations:** 1 General Surgery, The Brooklyn Hospital Center, Brooklyn, USA; 2 Radiation Oncology, Rutgers Robert Wood Johnson Medical School, New Brunswick, USA; 3 Clinical and Radiation Oncology, Shaukat Khanum Memorial Cancer Hospital and Research Centre, Lahore, PAK; 4 Radiology, Aziz Fatimah Medical and Dental College, Faisalabad, PAK

**Keywords:** rare location, rare disease, high dose radiotherapy, parapharyngeal space, epithelial-myoepithelial carcinoma

## Abstract

Epithelial-myoepithelial carcinoma (EMC) is a rare clinical entity that affects glandular tissues, most commonly salivary glands. EMC of parapharyngeal space is exceedingly rare. Surgery is the mainstay of treatment with or without chemotherapy, radiotherapy, or both. Due to the rarity of the disease, select cases where surgery is not possible present a management conundrum. We present a case of locally advanced, stage IVa EMC of parapharyngeal space that was treated with upfront definitive radiotherapy. Radiotherapy treatment alone led to long-term disease control in both clinical and radiological follow-ups. The patient was followed for more than eight years posttreatment with no disease recurrence, enjoying the normal activities of life with no late toxicities including xerostomia. This case report highlights the role of radiotherapy in the management of such patients, and more studies are required in this context for surgical candidates with positive disease margins.

## Introduction

Introduced in 1972, the term "epithelial-myoepithelial carcinoma (EMC)" refers to the low-grade malignant epithelial neoplasm that mainly affects salivary glands, accounting for ~1% of salivary gland tumors [1]. EMC affects both genders, with a slight female preponderance usually in their 50s and 60s. Histologically, two cell populations can be differentiated by immuno-histology analysis. Although EMC is a low-grade tumor and usually well-circumscribed, local recurrence and distant metastases are common, which can lead to poor prognosis [2,3]. EMC is primarily managed by surgery. Radiation therapy is indicated for positive or close margins. Due to its rarity, there are no clear guidelines for the management of locally advanced inoperable EMC [4]. We present a case of a 37-year-old male having locally advanced inoperable EMC of salivary glands of parapharyngeal space that was managed with definitive radiotherapy. The disease control was great following radiation therapy, and the patient was followed for eight consecutive years with no disease recurrence.

## Case presentation

A 37-year-old male, who is a smoker and diabetic, presented to the walk-in clinic in February 2015 with a history of swelling in the parotid region since childhood. There has been a gradual increase in the size of the swelling over the last four years. For the last two months, the patient complained of difficulty swallowing, tinnitus, and pressure effect in the right ear. The patient denied weight loss, loss of appetite, headache, cough, or a family history of cancer. On physical examination, there was a soft tissue mass with limited mobility starting at the temporomandibular joint over the parotid and extending below the angle of the mandible, separable from the submandibular gland and the mandible. No cervical lymphadenopathy was noted. Oral examination revealed pharyngeal swelling with convexity over the right side, and the posterior pharyngeal opening was restricted.

**Figure 1:**
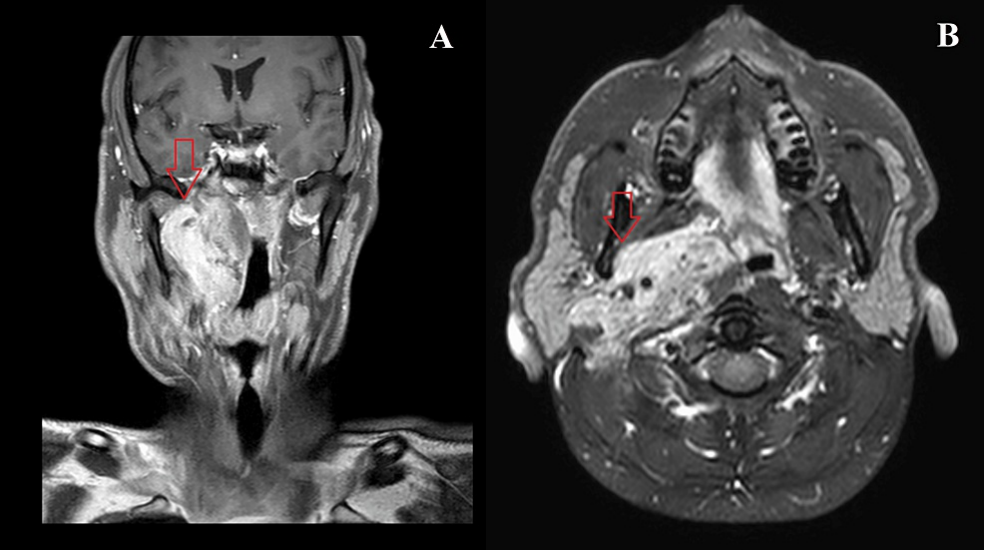
Magnetic resonance imaging of face and neck showing pretreatment images of the locally advanced tumor on the right side as indicated by the red arrows (A) Coronal view. (B) Axial view.

A tumor biopsy revealed nests of epithelial cells with moderate cytologic atypia and hyperchromatic nuclei. Spindle-shaped myoepithelial cells were present in the background. Immunohistochemical analysis was positive for p63. A diagnosis of EMC was made based on histology. Magnetic resonance imaging (MRI) of the face and neck demonstrated tumor enhancement in the right parapharyngeal space (9.2 x 5.5 x 2.5 cm in diameter) encasing the right carotid artery with intracranial extension (Figure 1).

There were bilateral sub-centimeter level II lymph nodes. A final diagnosis of poorly differentiated parapharyngeal carcinoma with stage T4 N2 M0 (Stage group IVA) and histology-type EMC was made.

**Figure 2:**
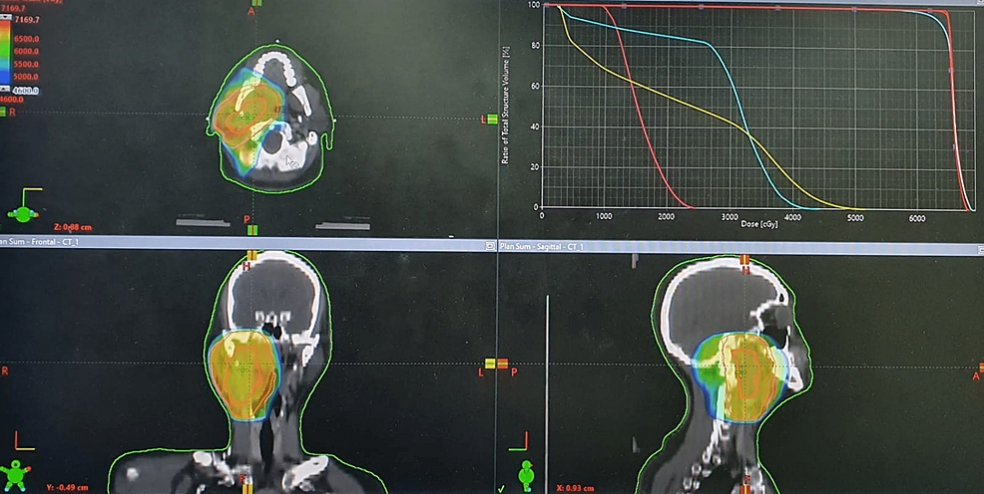
Multiplanar radiation therapy plan with dose constraints

The case was discussed in the multidisciplinary tumor board. The option of surgery was not indicated because of local invasiveness as the tumor was encasing the right carotid artery with intracranial extension as demonstrated on imaging. Hence, definitive radiotherapy was planned. Intensity-modulated radiation therapy technique was used to deliver 33 fractions of radiation therapy at a dose of 2 Gray per fraction daily from Monday to Friday, with Saturday and Sunday as off days (Figure 2). The patient received concurrent chemotherapy with tri-weekly Cisplatin 100 mg per meter square.

**Figure 3:**
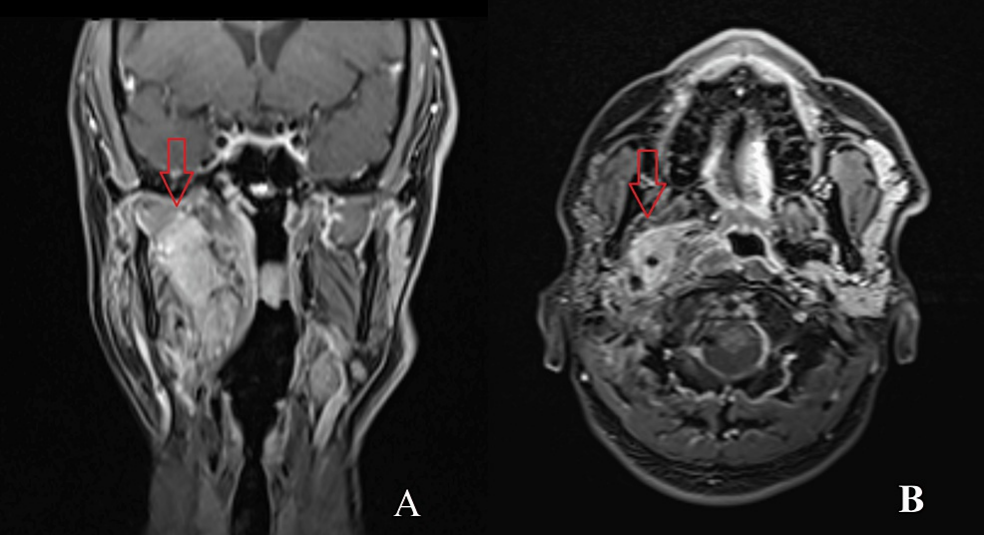
Magnetic resonance imaging showing posttreatment images of local tumor control eight years after treatment Red arrows show the treated disease site. (A) Coronal view. (B) Axial view.

The patient was followed weekly during radiation therapy; he tolerated the treatment well with only grade 1 mucositis toward the end of the treatment. He completed the treatment course as planned. After the completion of the treatment, the patient has been followed up with imaging, the latest of which shows disease control as shown in Figure 3. No long-term side effects especially xerostomia were observed in the patient on routine follow-up visits.

## Discussion

EMC is now accepted as a distinct entity despite various synonyms like clear cell adenoma, ductal salivary carcinoma, solid tubular adenoma, etc. [5]. Most of the individuals are in their fifth or sixth decade of life when first diagnosed with EMC with a slight female preponderance. It is exceedingly rare to find this tumor in children. In this study, we present a young male patient who describes that he has had this swelling for as long as he can remember. The parotid gland is the most common site of origin accommodating two-thirds of the cases; minor salivary glands come next with 10%-15%, and 10% have origin in the submandibular gland [1]. Clinical manifestations vary based on location and size. The parapharyngeal location of the tumor in our case caused dysphagia, tinnitus, and pressure symptoms in the ear. Despite being a low-grade tumor, reported local recurrence rates for EMC range from 23% to 50%, with 25% chances of distant metastasis [3].

Histological morphology may show different cell types like spindle, clear, rounded, epithelial, or plasmacytoid cells. Immunohistochemical analysis is helpful in making the diagnosis of positive reactivity toward p63 protein, S-100 protein, cytokeratin-7, and smooth muscle actin [6]. Usually, histopathological examination shows enlarged polygonal myoepithelial cells surrounding luminal ductal epithelial cells, a dual cell population from bidirectional stem cell differentiation. Histological features like cell types, stromal composition, immunoreactivity to different components, or patterns of growth did not indicate any difference in the clinicopathological behavior or disease prognosis, except for nuclear atypia.

Management of EMC is similar to other salivary gland tumors, primarily by surgery. Surgery can be followed by postoperative radiotherapy with or without chemotherapy in select cases where indicated. Cases where surgery is not an option, pose a treatment conundrum as there are no definite guidelines because of the rarity of the disease. Only one such case has been reported in which surgery was not an option and the disease was managed by upfront chemotherapy followed by radiotherapy [4]. Similarly, surgery was opted out in our case because of local invasiveness and intracranial extension of the disease. Radiotherapy with concurrent chemotherapy proved marvelous with disease-free survival for more than eight years on follow-up with imaging.

Certain factors are associated with aggressive behavior for EMC, which include positive margins, nuclear atypia, aneuploidy, the solid pattern of growth, high mitosis, and necrosis [3]. As high local recurrence and distant metastasis have been reported with this low-grade tumor, especially in patients with disease showing aggressive behavior, surgery alone may not be sufficient, and elective neck irradiation is warranted to prevent local recurrence. Further studies are needed for better prospects of such cases as the number of cases being reported is increasing.

## Conclusions

EMC is a rare disease, with reports of cases on the rise. Treatment guidelines are not clear-cut in cases where surgery cannot be employed. Radiotherapy can be a valuable and effective treatment modality in such cases. Pending further studies, the significance of radiotherapy as a primary treatment option can be made use of.
